# Comparison of Antimicrobial Resistance Detected in Environmental and Clinical Isolates from Historical Data for the US

**DOI:** 10.1155/2020/4254530

**Published:** 2020-04-11

**Authors:** Megan Hua, William Huang, Albert Chen, Michael Rehmet, Cade Jin, Zuyi Huang

**Affiliations:** Department of Chemical Engineering, Villanova University, Villanova, PA 19085, USA

## Abstract

Antimicrobial resistance (AMR) has become an urgent public health issue, as pathogens are becoming increasingly resistant to commonly used antimicrobials. While AMR isolate data are available in the NCBI Pathogen Detection Isolates Browser (NPDIB) database, few researches have been performed to compare antimicrobial resistance detected in environmental and clinical isolates. To address this, this work conducted the first multivariate statistical analysis of antimicrobial-resistance pathogens detected in NPDIB clinical and environmental isolates for the US from 2013 to 2018. The highly occurring AMR genes and pathogens were identified for both clinical and environmental settings, and the historical profiles of those genes and pathogens were then compared for the two settings. It was found that *Salmonella enterica* and *E. coli* and *Shigella* were the highly occurring AMR pathogens for both settings. Additionally, the genes *fosA*, *oqxB*, *ble*, *floR*, *fosA7*, *mcr-9.1*, *aadA1*, *aadA2*, *ant(2”)-Ia*, *aph(3”)-Ib*, *aph(3')-Ia*, *aph(6)-Id*, *blaTEM-1*, *qacEdelta1*, *sul1*, *sul2*, *tet(A)*, and *tet(B)* were mostly detected for both clinical and environmental settings. Ampicillin, ceftriaxone, gentamicin, tetracycline, and cefoxitin were the antimicrobials which got the most resistance in both settings. The historical profiles of these genes, pathogens, and antimicrobials indicated that higher occurrence frequencies generally took place earlier in the environmental setting than in the clinical setting.

## 1. Introduction

Every year, foodborne pathogens cause the sicknesses of one in six Americans, hospitalize 128,000, and cause the deaths of 3,000 [1]. There are 31 known pathogens that cause foodborne illness, and several unspecified agents that have yet to be identified [2, 3]. Unfortunately, these pathogens obtain resistance to commonly used antimicrobials [4]. In order to combat AMR foodborne pathogens, the National Antimicrobial Resistance Monitoring System (NARMS) has been gathering data from human clinical samples, animal slaughter samples, and retail meat samples [5, 6]. It allows people to investigate the resistance phenotypes of pathogens to specific antimicrobials over time. In addition, the National Database of Antibiotic Resistant Organisms (NDARO) has been collecting genetic and antibiotic susceptibility data, which are posted in the NCBI Pathogen Detection Isolates Browser (NPDIB). In particular, NPDIB contains useful information on AMR pathogen isolates throughout the countries in the world, including sampling locations, date, isolation sources, isolate types, and antimicrobial-resistance genotypes. Useful information on antimicrobial resistance should be obtained by analyzing the data from NPDIB.

While programs like AMRFinderPlus [7] have been developed to identify AMR genes in bacterial genomes, AMR genes and pathogens from the environmental and clinical isolates in NPDIB have not been thoroughly compared. Only few data analysis studies have been conducted on the data in NPDIB. In one of these studies, genome sequences of *Salmonella enteritidis* isolates from mice that were caught on Pennsylvania poultry farms in the mid-1990s were compared to identify frequently encountered mutations in the evolution of isolates [8]. Another study compared the whole genome sequences of *Salmonella Heidelberg* strains isolated from feces of Canadian broiler chickens with those from NPDIB to identify AMR genes [9]. Similarly, Reference [10] used NPDIB data to identify AMR genes and cassettes in *Salmonella enteritidis* isolated from sources associated with the United States food animals between 1998 and 2011. Reference [11] utilized NPDIB data to determine whether San Diego coastal waters contained pathogenic bacteria by genome sequence comparison. In another analysis that was based upon the NPDIB database, the impact from meat sources and locations on antimicrobial resistance in foodborne pathogens across six US. states (i.e., PA, NY, MD, NM, MN, and CA) was studied [12]. It was found that chicken and turkey were the two major meats that carried AMR genes in the six US states. It was concluded that geographically distinct sites did not have substantially different AMR gene prevalence due to the carrying of those genes by chicken and turkey across the country [12]. However, the trend of the occurrence of neither AMR genes nor pathogens was studied in that study. Reference [13] addressed this issue by investigating the trends of antimicrobial resistance in foodborne pathogens in eight countries from the historical sample data in the NPDIB database. That study indicated that the overall number of reported antimicrobial-resistance cases in foodborne pathogens is generally rising overtime in the eight countries (including the US, the UK, China, Brazil, Mexico, Canada, Australia, and South Africa). While References [12, 13] were mainly focused on the genes detected from foodborne pathogens, it is necessary to study antimicrobial resistance shown in clinical pathogens. Reference [14] investigated antimicrobial-resistance genes in historical clinical samples from the NPDIB database for six different countries from majorly inhabited landmasses, including Australia, Brazil, China, South Africa, the UK, and the US. It was found that several of these significant genes (i.e., *aph*(3”)*-Ib*, *aph*(6)*-Id*, *blaTEM-*1, and *qacEdelta1*) are shared among all six countries studied. The most shared pathogens responsible for carrying the most important genes in the six countries in the clinical setting were *Acinetobacter baumannii*, *E. coli* and *Shigella*, *Klebsiella pneumoniae*, and *Salmonella enterica*. South Africa carried the least similar antimicrobial genes to the other countries in clinical isolates. Since the aforementioned studies were focused on either foodborne or clinical pathogens, further comparison of the genes sampled from environmental and clinical isolates is necessary to study the similarity of AMR genes and pathogens in these two settings. As suggested by a study done in 1997 [15], the genetic element coding for vancomycin resistance in *Enterococcus faecium* isolated from livestock was also found in humans, which indicated the possibility of gene transfer between related organisms from these different settings (i.e., livestock versus humans).

In this study, around 8,000 NPDIB isolate samples for the US from 2013 to 2018 were analyzed to study the relationship between AMR genes in pathogens in environmental and clinical settings. This study was focused on the data for the US, as the US is the only country that provides antimicrobial susceptibility testing phenotypes, especially after 2013. Different from the aforementioned studies, the NPDIB data was analyzed to a greater extent in this study. In particular, we compared the genes, pathogens, and antimicrobial susceptibility between the environmental and clinical samples that showed resistance to at least one antimicrobial. In addition, the trend of the occurrences of both clinical and environmental AMR genes was studied for those AMR isolates. Since the dataset contained almost 8,000 samples, a multivariate statistical approach named principal component analysis (PCA) [16, 17] was used to visualize the multidimensional data in a two-dimensional space. Additionally, hierarchical clustering trees [18–20] were used to group similar objects (e.g., AMR genes, pathogens, and antimicrobials) into separate clusters to create a dendrograms for studying the correlations between AMR pathogens and genes in the clinical and environmental settings.

## 2. Results

### 2.1. Comparing AMR Pathogens Found in Environmental and Clinical Samples

**Figure 1:**
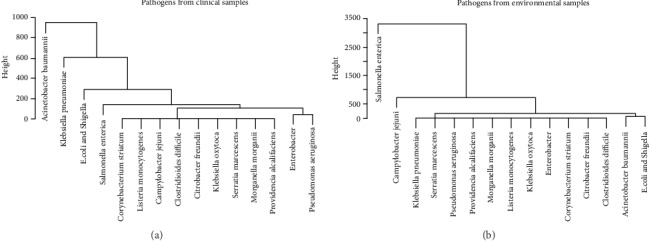
Hierarchical clusters of pathogens from clinical and environmental samples based on the number of detected AMR genes: (a) pathogens from clinical samples and (b) pathogens from environmental samples.

**Figure 2:**
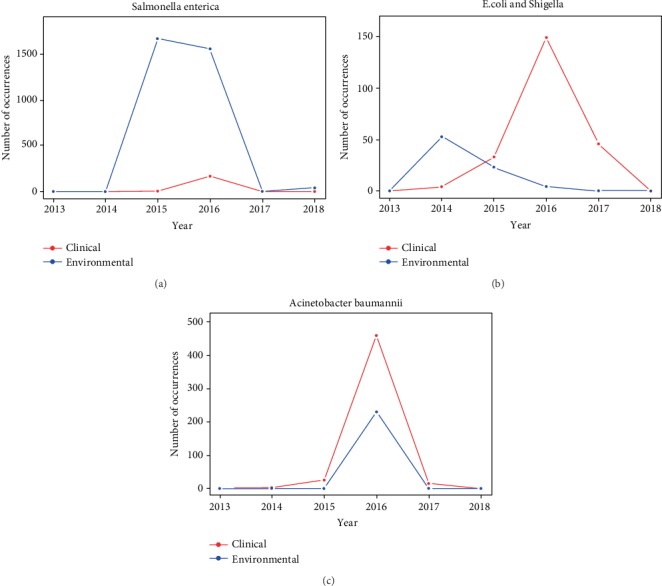
Number of antimicrobial resistance occurrences from 2013 to 2018 for major pathogens: (a) *Salmonella enterica*, (b) *E. coli* and *Shigella*, and (c) *Acinetobacter baumannii*.

A matrix was built in which each row represented one sampled pathogen and each column stood for one gene. Each element in the matrix represented the number of samples in which the pathogen in the row and the gene in the column were detected at the same time. Major pathogens were detected from the hierarchical clustering result (Figure 1), along with the visualization of the pathogens in the two-dimensional space created from principal component analysis (Supplementary [Supplementary-material supplementary-material-1]). The clusters for environmental and clinical isolates were compared to each other to identify major pathogens that exhibited the most resistance in both settings. Pathogens carrying a larger amount of AMR genes were typically located on the top branches in the clustering trees and separated from the larger groups. The major pathogens identified from clinical isolates included *Acinetobacter baumannii*, *Klebsiella pneumoniae*, *E. coli* and *Shigella*, *Salmonella enterica*, *Enterobacter*, and *Pseudomonas aeruginosa*, while those mostly found in environmental samples were *Salmonella enterica*, *Campylobacter jejuni*, *Acinetobacter baumannii*, and *E. coli* and *Shigella*. To further compare the pathogens detected in clinical and environmental samples, the number of yearly AMR isolates for those major pathogens that were detected in both clinical and environmental settings from 2013 to 2018, including *Salmonella enterica*, *Acinetobacter baumannii*, and *E. coli* and *Shigella*, is graphed in Figure 2.

It is interesting to see that occurrence frequencies in pathogens from environmental samples were generally higher than those from the clinical samples for the same species, especially shown in historical occurrence profiles of *Salmonella enterica* and *E. coli* and *Shigella* in Figure 2. *Salmonella enterica* is a bacillus, gram-negative bacteria.  Figure 2(a) shows that from 2013 to 2014, there were few occurrences of antimicrobial resistance in the clinical or environmental setting for this pathogen. From 2015 to 2016, however, there was a drastic increase in environmental isolates of over 1500 occurrences each year while the clinical isolates only increased slightly in 2016. In 2017 and 2018, both settings saw a drop to a low number of isolates. Compared to *Salmonella enterica*, *E. coli* and *Shigella* showed slightly different trends. These two pathogens are gram-negative bacteria commonly found in the gut [21]. While most strains are harmless, there are a few that can cause major infections. These strains are commonly found in contaminated, uncooked food and water.  Figure 2(b) shows that there was first an increase to over 50 AMR isolates in the environmental setting in 2014 with only a slight increase in clinical isolates in that same year. After 2014 and into 2018, however, the number of environmental AMR isolates gradually decreases to close to none. In the clinical setting, there was a continuous rise in isolates with a maximum of about 150 isolates in 2016, but then a fall in 2017 and 2018. While the trend shown in the clinical setting looks like the one in the environmental setting, there was a time delay between the most occurring years for these two settings. Different from the historical profiles of the other two pathogens, the profiles of *Acinetobacter baumannii*, which is bacillus, gram-negative bacteria typically found in clinical conditions [8], showed little time delay between the environmental and clinical settings in the occurrence frequency (Figure 2(c)).

### 2.2. Comparing Antimicrobial-Resistance Genes Found in Environmental and Clinical Samples

**Figure 3:**
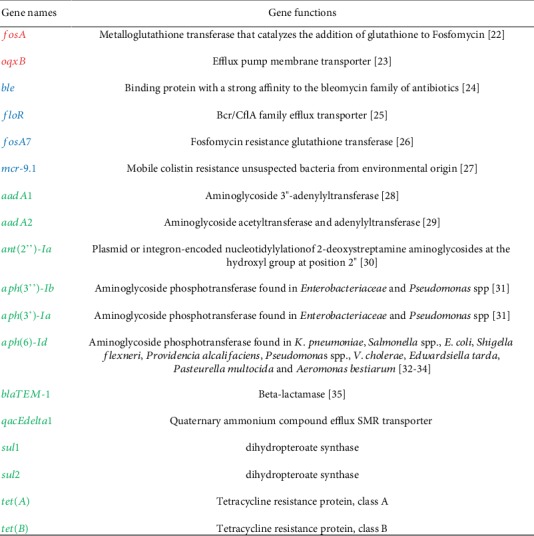
The functions of the major AMR genes identified from the US isolate dataset (Red: AMR genes mostly found in clinical isolates; Blue: AMR genes mostly found in environmental isolates; and Green: AMR genes commonly found in both clinical and environmental settings).

Similar to the previous section, PCA and hierarchical clustering were used to identify the genes that contributed the most to the resistance of antimicrobials and were compared across clinical and environmental isolates. Genes mostly detected in samples were separated from the larger groups of genes on the top branches of the clustering tree (Supplementary Figure 2). Upon comparing the most common AMR genes in clinical isolates to those in environmental isolates, the following major genes were categorized as AMR genes mostly found in clinical isolates (*fosA* and *oqxB*), AMR genes mostly found in environmental isolates (*ble*, *floR*, *fosA7*, and *mcr-9.1*), and AMR genes commonly found in both clinical and environmental settings (*aadA1*, *aadA2*, *ant*(*2”*)*-Ia*, *aph*(*3”*)*-Ib*, *aph*(*3'*)*-Ia*, *aph*(*6*)*-Id*, *blaTEM-1*, *qacEdelta1*, *sul1*, *sul2*, *tet*(*A*), and *tet*(*B*)). Categorizing the aforementioned genes into different groups was based upon the occurring frequencies in the analysis of the NPDIB data for the US. It did not exclude the detection of the gene from one setting in the other setting. For example, although *fosA7* was detected in more environmental isolates in this study, it was also detected in human clinical cases. Since this work is aimed at identifying genes mostly involved in antimicrobial resistance, instead of providing thorough investigation of the functions of those genes, a brief introduction of each of the aforementioned genes is given in Figure 3 [22–35].

**Figure 4:**
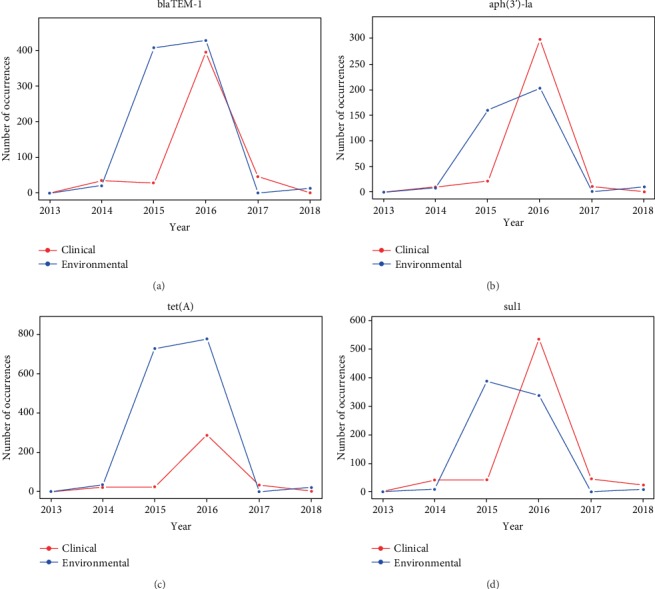
Yearly profiles of selected representative major genes: (a) *blaTEM-1*, (b) *aph*(3')*-la*, (c) *tet*(*A*), (D) *sul1*.

All AMR genes listed in Figure 3 were further analyzed with their historical occurrence profiles. Through these profiles, the results for environmental and clinical settings were compared across the years. Due to the space constraint, only four representative genes from the clinical-environmental common group, including *blaTEM-1*, *aph*(3')*-la*, *tet*(*A*), and *sul1*, are chosen to be plotted in Figure 4. These genes were chosen to compare the trends shown in the AMR gene profiles for the clinical and environmental settings. The profiles for other genes can be found in Supplementary [Supplementary-material supplementary-material-1]. It can be seen from Figure 4 that AMR genes *blaTEM-1*, *aph*(3')*-la*, *tet*(*A*), and *sul1* are all detected in environment isolates more often than in clinical isolates in the earlier years.

### 2.3. Comparing Antimicrobials with Resistance Detected in Clinical and Environmental Samples

**Figure 5:**
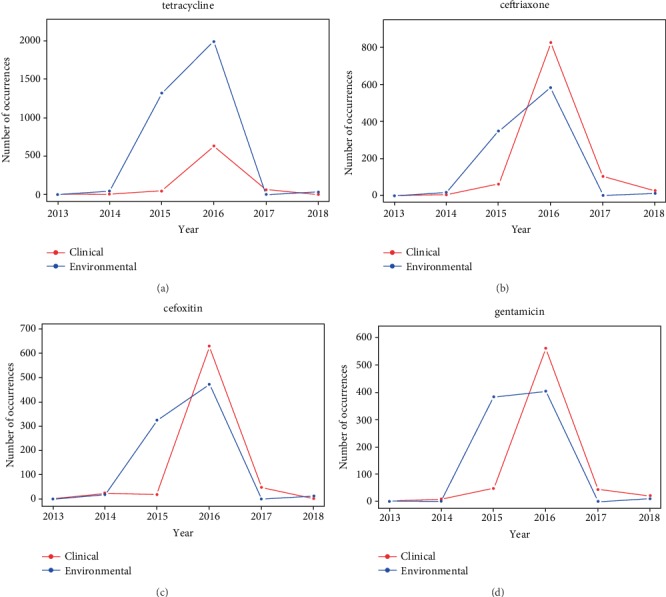
Yearly profiles of selected major antimicrobials: (a) tetracycline, (b) ceftriaxone, (c) cefoxitin, and (d) gentamicin.

Similar to the previous sections, PCA and hierarchical cluster were used to identify antimicrobials with high resistance frequencies in clinical and environmental isolates (Supplementary [Supplementary-material supplementary-material-1]). The major antimicrobials that got high resistance frequencies to clinical and environmental isolates were categorized as antimicrobials mostly detected in clinical isolates with resistance (ciprofloxacin, trimethoprim-sulfamethoxazole, cefazolin, ceftazidime, aztreonam, levofloxacin, nitrofurantoin, amikacin, ampicillin sulbactam, tobramycin, and cefotaxime), antimicrobials mostly detected in environmental isolates with resistance (ceftiofur, amoxicillin-clavulanic acid, kanamycin, streptomycin, and sulfisoxazole), and antimicrobials commonly detected in both clinical and environmental isolates with resistance (ampicillin, ceftriaxone, gentamicin, tetracycline, and cefoxitin). The antimicrobials in the same category generally show similar historical occurrence profiles. Four antimicrobials commonly detected in both clinical and environmental isolates were selected to compare the trend of the antimicrobials that got resistance in the clinical and environmental settings, while the occurrence profiles for other antimicrobials can be found in Supplementary [Supplementary-material supplementary-material-1]. In particular, the selected antimicrobials include tetracycline (used in both clinical and environmental settings but often in subtherapeutic doses on livestock [36]), ceftriaxone (a broad spectrum cephalosporin that is mainly used against gram-positive bacteria to treat conditions, such as lower respiratory tract, skin, and urinary tract infections [37]), cefoxitin (a broad-spectrum cephalosporin used to treat bacterial infections, such as pneumonia, blood infections, and abdominal infections [38]), and gentamicin (an aminoglycoside antibiotic that is used to treat infections, such as meningitis, blood infections, and urinary tract infections [39]). The yearly occurrence samples of these antimicrobials are plotted in Figure 5, which shows that the occurrence of resistance to antimicrobials detected with resistance in environmental isolates generally had more cases than the antimicrobials detected with resistance in clinical isolates in earlier years.

## 3. Discussion

### 3.1. Environmental Isolates Led Clinical Isolates with Higher AMR Occurrence Cases in an Earlier Time

The most common trend that is found in Figures 2 and 4 was that the AMR genes and pathogens were detected more frequently in environmental samples than in clinical isolates in earlier years, followed by a dramatical increase in the detection in the number of clinical isolates one or two years later. For example, the number of environmental isolates that contained gene *sul1* increased to nearly 400 in 2015, while the number of clinical isolates with that gene only increased slightly in the same year. In 2016, clinical isolates surged and even surpassed the number of environmental isolates. Similarly, many pathogens followed a similar trend of an increase in environmental isolate samples followed by an increase in clinical isolate samples. For example, the number of environmental isolates *E. coli* and *Shigella* peaked in 2014, while clinical isolates peaked in 2015 and increased even more in 2016.

This overall trend may be caused by the passing of these AMR genes from the environmental setting to the clinical setting by microorganisms, such as through infections acquired from environmental sources (e.g., by *Salmonella enterica* and *E. coli* and *Shigella*). Cases on the gene transfer from food animals to humans have been reported (see Reference [40, 41] for examples). However, the trend shown in the results is not the direct evidence of the AMR gene transfers from the environmental isolates to clinical isolates. It only shows the correlation of the occurrence frequency of AMR genes and pathogens between the clinical and environmental isolates. In order to prove the genes shown in Figure 4 were transferred from environmental isolates to clinical isolates, extensive comparison of genomic sequences of the related samples from the 8,000 isolates would be needed. In addition, detailed analysis of the isolate time, sources, and locations would be required to identify the paths for those genes to transfer from the environmental setting to the clinical setting. While this study did not have space for exploring all the aforementioned work, it did provide useful information to direct those future work.

### 3.2. Antimicrobial Resistance and the Overuse of Antimicrobials

While thousands of pathogen isolates were reported from 2013 and 2019 for the US, this study was only focused on the isolates with tested resistance to at least one known antimicrobial. One hypothesis behind this was that the occurrence frequencies of AMR isolates had certain correlation with the consumption of antimicrobials. It was interesting to see that the occurrence frequencies of AMR genes and pathogens generally increased in the first few years and began to decrease after 2016. One of the potential reasons for the increasing antimicrobial resistance in 2013 to 2016 is the overuse of antimicrobials in raising animals. It was reported that around 80% of all antimicrobials sold in the US were used in animal agriculture and approximately 70% of these animal-used antimicrobials were those used as human medicine [42]. The CDC found that beta-lactam use had increased by 26%, carbapenems by 37%, and third- and fourth-generation cephalosporins by 12% from 2006 to 2012. When antibiotics are overused, vulnerable bacteria are killed; however, more resistant pathogens can survive. These resistant bacteria then multiply causing the amount of antimicrobial-resistance bacteria to rapidly increase, as shown in Figures 2–5 for years 2013 to 2016. On the other hand, the occurrence frequencies for most pathogens and genes in 2017 showed a decreasing trend in Figures 2 and 4. According to the FDA, domestic sales and distribution of medically important antibiotics in livestock decreased by 33% from 2016 to 2017 [43]. This is a potential reason to explain the overall low number of AMR isolates in 2017.

### 3.3. Limitation of This Study

While the data from NPDIB was carefully studied to identify the relationship between the AMR genes and pathogens detected in environmental and clinical samples, there are possible sources of errors that should be noted. First, the NPDIB may be incomplete, as hospitals and other medical centers were voluntary to record their isolates and they might not input each isolation into the NPDIB database. In 2013 and 2014, there were only a small amount of recorded isolates of antimicrobial resistance. It is unknown whether there were actual low levels of antimicrobial resistance or not many hospitals reported all isolates. The latter may be most likely true. Despite this, the NPDIB is still a large, comprehensive dataset. In addition, if a hospital did not report all isolates, this should be applicable to both clinical and environmental settings. Therefore, the trends shown in Figures 2–5 should be still valid.

In order to account for the variation in the number of submitted isolates each year, normalized profiles of the major genes and pathogens were made. Normalized data for each year was obtained by dividing the number of AMR isolates for individual major genes/pathogens by the total number of AMR samples collected in that year. In this way, the percent of the total number of resistance isolates in each year that involved a specific gene/pathogen could be calculated. However, because this study was mainly focused on comparing data between the clinical and environmental isolates for the same time periods, the different number of submitted samples over years did not affect the comparing results. For example, people obtained more environmental samples in 2016 than in 2013, due to the increased awareness of antimicrobial resistance and the development of microbial/gene detection techniques. Similarly, more clinical samples were obtained in 2016 than in 2013.

It was reported by Zhang et al. [12] that geographically distinct sites (i.e., PA, NY, MD, CA, MN, NM) did not have substantially different AMR gene prevalence. In addition, a certain amount of samples (around 10% of the total samples) did not contain any geographic location information. This study thus did not study the correlation of antimicrobial resistance between the clinical and environmental isolates for individual states in the US. However, further research is suggested for detailed genome comparison for local clinical and environmental isolates to investigate the geographically specific AMR correlation for individual states in the US.

## 4. Materials and Methods

### 4.1. The Antimicrobial Data from the NCBI Pathogen Detection Isolates Browser

The antimicrobial resistance data was extracted from the NCBI Pathogen Detection Isolates Browser, which was created to identify sources of potential food contamination for the purpose of investigating foodborne diseases. The data from the NCBI browser was organized into a matrix. Each row of the matrix represented an isolate sample, and each column contained the following information about a variable of the corresponding sample: (1) the pathogen name, (2) the collection year, (3) whether the sample was clinical or environmental (the number 1 representing a clinical sample and 2 standing for an environmental sample), (4) the number of antimicrobials resisted by the pathogen in the sample, (5) the number of antimicrobials used in the susceptibility test, (6) the number of AMR genes detected, and (7) the collection location/state, (8-80) whether a specific gene was detected in the pathogen (the number 0 indicating that the gene was not detected in the sample and 1 meaning the detection of the gene in the column), (81-153) whether a specific antimicrobial was resisted (the number 0 indicating that the antimicrobial was effective against the pathogen in the sample and 1 meaning that the antimicrobial was resisted), and (154-157) if the samples were taken from enviromental, the meat source (i.e., chicken, turkey, beef, and pork).

### 4.2. Principal Component Analysis and Hierarchical Clustering

**Figure 6:**
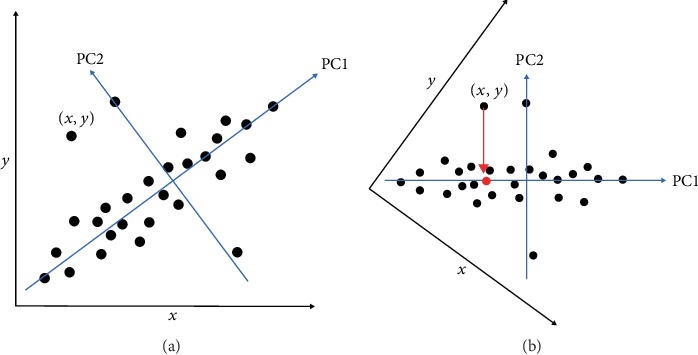
Illustration of PCA: the data point (*x*, *y*) in the *x*~*y* coordinate space (a) is represented by its projection onto the PC1 direction (b). The two-dimensional space (i.e., *x*~*y*) is reduced to one dimension (i.e., PC1).

The data matrix was of around 8,000 rows (i.e., samples) and 157 columns (i.e., dimensions). It is impossible to project this high-dimensional data onto a two-dimensional space without using dimension reduction techniques like principal component analysis (PCA).  Figure 6 illustrates how to reduce the **x**~**y** space (two dimensions) into the PC1 space (one dimension) by rotating the coordinate system. For example, the (**x**, **y**) point in Figure 6(a) can be represented by its projection (the point in RED) onto PC1 in Figure 6(b). PC1 is the first principal component, a direction onto which the projections have the largest variance, to retain the most information contained in the data. The second principal component (PC2), which is orthogonal to PC1, has the second largest variance in its projections. In this work, the high-dimensional data was visualized on the PC1~PC2 space.

PCA allows for the visualization of the similarities and differences of different data points in a two-dimensional space. However, the projects of the large amount of objects (e.g., AMR genes, pathogens, and antimicrobials) are typically too crowded to distinguish. The hierarchical clustering approach was further used in this work to separate the studied objects. In hierarchical clustering, data points in the same clusters had similar properties and points grouped together lower in the clustering tree were more similar to each other than those that were grouped higher in the tree.

After plotting the data for both clinical and environmental genes and pathogens into the format of the hierarchical clustering tree, the important AMR genes and pathogens were identified. The PCA plots were made into clustering trees that allowed for easy recognition of the genes and pathogens, making comparison of the clinical and environmental results easier. The historical number of occurrences of samples that contained the important pathogens, genes, and antimicrobials was plotted for 2013 to 2018. Those historical occurrence profiles begin in 2013 because antimicrobial susceptibility information became available in that year. Those profiles were then used to study the trend of antimicrobial resistance in clinical and environmental pathogens. In the graphs for important pathogens, the **x**-axis represents the years while the **y**-axis represents the number of pathogens discovered with antimicrobial resistance. In the graphs for important genes, the **y**-axis shows the number of occurrences the gene was discovered in a pathogen. In the antimicrobial graphs, the **y**-axis represents the number of instances antimicrobial resistance was found for that specific antimicrobial. The similarities between the graphs were studied to determine whether there was a correlation between clinical and environmental antimicrobial resistance.

## 5. Conclusion

It is important to study the antimicrobial resistance in both clinical and environmental settings. In this work, approximately 8,000 AMR pathogen samples for the US from NCBI Pathogen Detection Isolates Browser were analyzed by multivariate statistical methods to visualize high-dimensional data in two-dimensional space and identify pathogens, AMR genes, and antimicrobials that were mainly involved in microbial resistance. The yearly profiles of the occurrence of pathogens, AMR genes, and antimicrobials were further analyzed for both clinical and environmental isolates. The results indicated that the most common AMR pathogens for both clinical and environmental settings were *E. coli* and *Shigella* and *Salmonella enterica*; the genes mostly involved in antimicrobial resistance were *fosA*, *oqxB*, *ble*, *floR*, *fosA7*, *mcr-9.1*, *aadA1*, *aadA2*, *ant(2”)-Ia*, *aph(3”)-Ib*, *aph(3')-Ia*, *aph(6)-Id*, *blaTEM-1*, *qacEdelta1*, *sul1*, *sul2*, *tet*(*A*), and *tet*(*B*); the antimicrobials to which pathogens were most resistant to in both clinical and environmental settings were ampicillin, ceftriaxone, gentamicin, tetracycline, and cefoxitin.

The historical profiles indicated AMR genes and pathogens showed higher occurrence frequencies in the environmental setting than in the clinical setting in early years (i.e., 2013-2015). Additionally, *Salmonella enterica* and *E. coli* and *Shigella* contained the most antimicrobial-resistance genes among all major pathogens, implying that they were most likely the main spreaders of resistance genes through horizontal gene transfer. Finally, it was concluded that the increased usage of unnecessary antimicrobials was correlated to an increase in antimicrobial resistance in pathogens. This work thus provided a data-driven evidence for the gene transfer between humans and livestock.

This study was focused on the antimicrobial resistance in the US due to the lack of antimicrobial susceptibility data in other countries. Future research should be conducted in other parts of the world to observe differences in antimicrobial resistance across the globe. Such research will not only raise awareness about the rising issue of antimicrobial resistance, but it will also allow national organizations, such as the United States Department of Agriculture, to develop new regulations on antimicrobial usage in agriculture and help alleviate the AMR problem.

## Data Availability

If the manuscript gets accepted, the data may be available upon email request.

## Supplementary Materials

Supplementary Materials Supplementary Figure 1: the projection of the pathogens onto the two-dimensional space characterized by Principal Component 1 (PC1) and Principal Component 2 (PC2): (A) clinical samples and (B) environment samples. The outlier pathogens were found important in carrying antimicrobial-resistance (AMR) genes. Those overlapping pathogens, which were further distinguished via hierarchical clustering (Figure 1), were not as important from the perspective of occurrences and AMR gene carrying. Supplementary Figure 2: (A) the clustering of clinical AMR genes; (B) the projection of clinical AMR genes onto the two-dimensional space characterized by PC1 and PC2; (C) the clustering of environmental AMR genes; (D) the projection of environmental AMR genes onto the two-dimensional space characterized by PC1 and PC2. Supplementary Figure 3: the historical occurrence profiles of the other AMR genes (excluding the genes shown in Figure 4). Supplementary Figure 4: (A) the clustering of antimicrobials resisted by clinical isolates; (B) the projection of clinical antimicrobials onto the two-dimensional space characterized by PC1 and PC2; (C) the clustering of antimicrobials resisted by environmental isolates; (D) the projection of environmental antimicrobials onto the two-dimensional space characterized by PC1 and PC2. Supplementary Figure 5: the historical occurrence profiles of antimicrobials resisted by clinical and environmental isolates.
