# Supplementary material for: Comparison of Antimicrobial Resistance Detected in Environmental and Clinical Isolates from Historical Data for the US
Source: Biomed Res Int. 2020 Apr 11;2020:4254530. doi: 10.1155/2020/4254530 (PMC7174961; doi:10.1155/2020/4254530)
Supplement: Supplementary Materials — Supplementary Figure 1: the projection of the pathogens onto the two-dimensional space characterized by Principal Component 1 (PC1) and Principal Component 2 (PC2): (A) clinical samples and (B) environment samples. The outlier pathogens were found important in carrying antimicrobial-resistance (AMR) genes. Those overlapping pathogens, which were further distinguished via hierarchical clustering (Figure 1), were not as important from the perspective of occurrences and AMR gene carrying. Supplementary Figure 2: (A) the clustering of clinical AMR genes; (B) the projection of clinical AMR genes onto the two-dimensional space characterized by PC1 and PC2; (C) the clustering of environmental AMR genes; (D) the projection of environmental AMR genes onto the two-dimensional space characterized by PC1 and PC2. Supplementary Figure 3: the historical occurrence profiles of the other AMR genes (excluding the genes shown in Figure 4). Supplementary Figure 4: (A) the clustering of antimicrobials resisted by clinical isolates; (B) the projection of clinical antimicrobials onto the two-dimensional space characterized by PC1 and PC2; (C) the clustering of antimicrobials resisted by environmental isolates; (D) the projection of environmental antimicrobials onto the two-dimensional space characterized by PC1 and PC2. Supplementary Figure 5: the historical occurrence profiles of antimicrobials resisted by clinical and environmental isolates. [file 4254530.f1.docx]

Legends for Supplementary Figures

Supplementary Figure 1: the projection of the pathogens onto the two-dimensional space characterized by Principal Component 1 (PC1) and Principal Component 2 (PC2): (A) clinical samples; (B) environment samples. The outlier pathogens were found important in carrying antimicrobial-resistance (AMR) genes. Those overlapping pathogens, which were further distinguished via hierarchical clustering (Figure 1), were not as important from the perspective of occurrences and AMR gene carrying.

Supplementary Figure 2: (A) the clustering of clinical AMR genes; (B) the projection of clinical AMR genes onto the two-dimensional space characterized by PC1 and PC2; (C) the clustering of environmental AMR genes; (D) the projection of environmental AMR genes onto the two-dimensional space characterized by PC1 and PC2.

Supplementary Figure 3: the historical occurrence profiles of the other AMR genes (excluding the genes shown in Figure 3).

Supplementary Figure 4: (A) the clustering of antimicrobials resisted by clinical isolates; (B) the projection of clinical antimicrobials onto the two-dimensional space characterized by PC1 and PC2; (C) the clustering of antimicrobials resisted by environmental isolates; (D) the projection of environmental antimicrobials onto the two-dimensional space characterized by PC1 and PC2.

Supplementary Figure 5: the historical occurrence profiles of antimicrobials resisted by clinical and environmental isolates.

Supplementary Materials


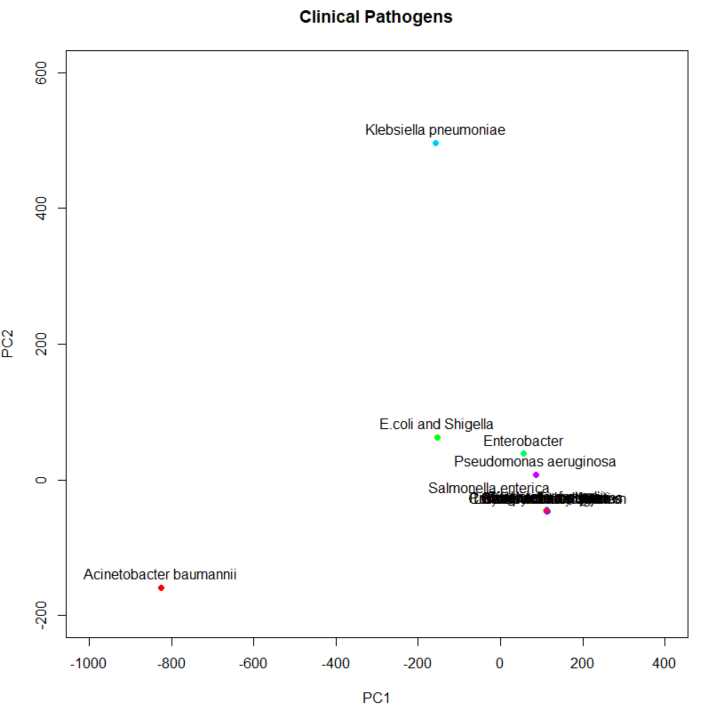

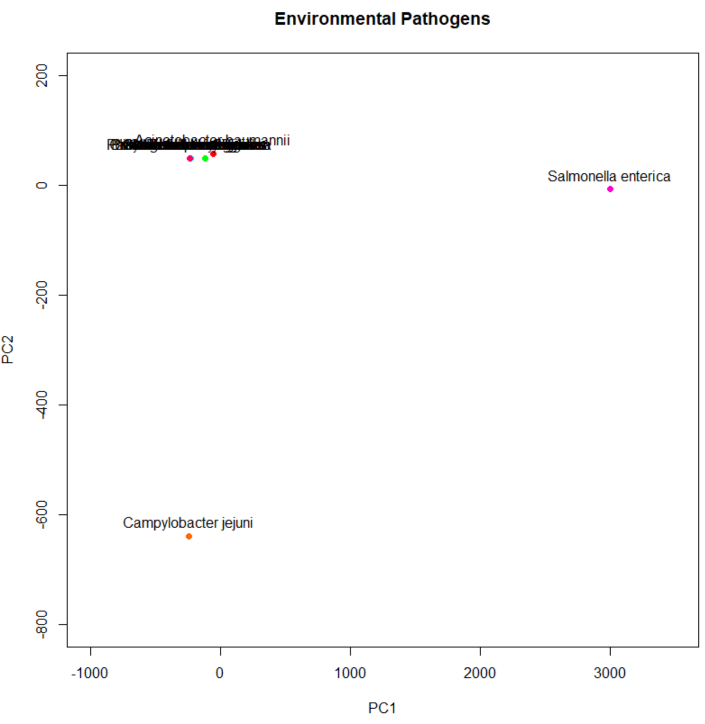


1. (B)

Supplementary Figure 1: the projection of the pathogens onto the two-dimensional space characterized by Principal Component 1 (PC1) and Principal Component 2 (PC2): (A) clinical samples; (B) environment samples. The outlier pathogens were found important in carrying antimicrobial-resistance (AMR) genes. Those overlapping pathogens, which were further distinguished via hierarchical clustering (Figure 1), were not as important from the perspective of occurrences and AMR gene carrying.


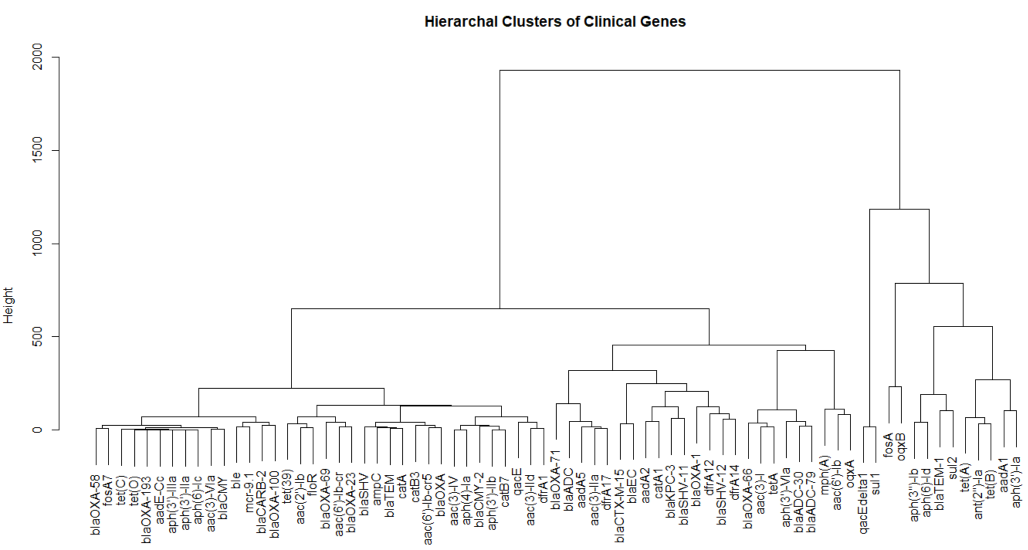


(A)


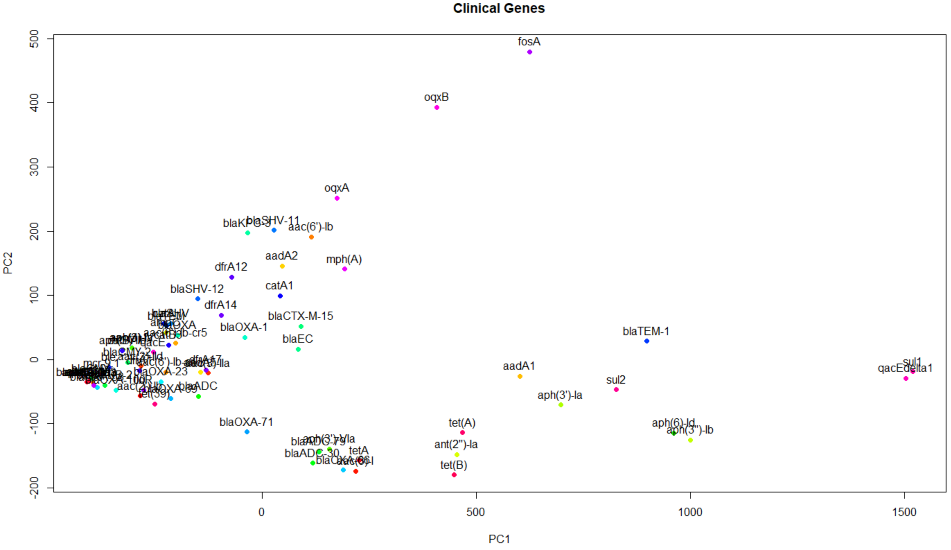


(B)


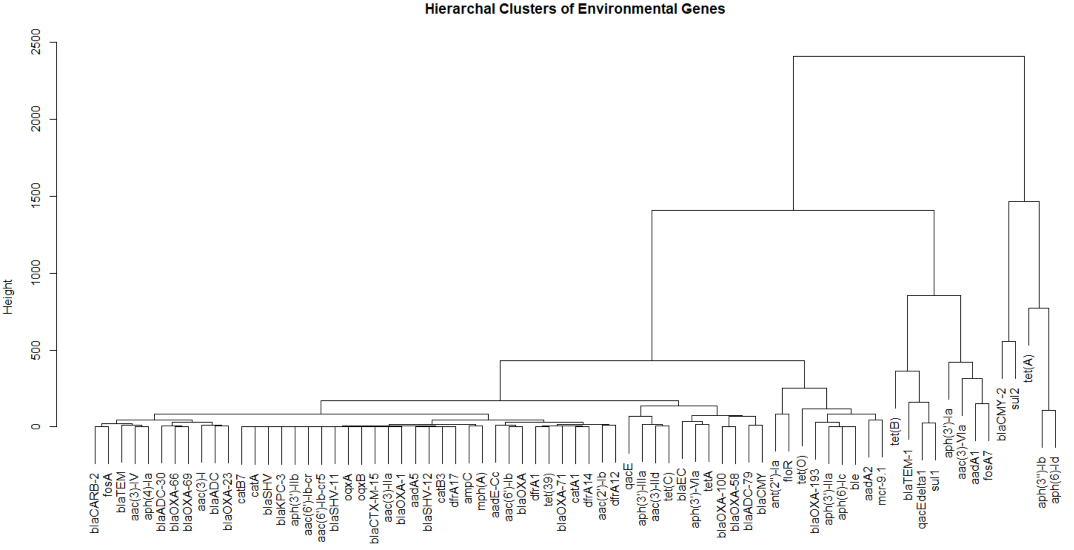


(C)


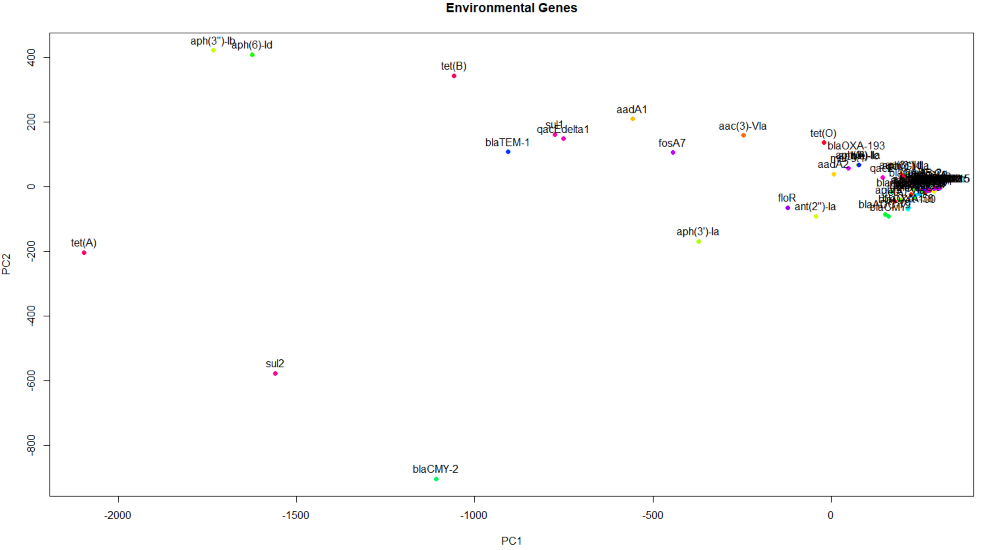


(D)

Supplementary Figure 2: (A) the clustering of clinical AMR genes; (B) the projection of clinical AMR genes onto the two-dimensional space characterized by PC1 and PC2; (C) the clustering of environmental AMR genes; (D) the projection of environmental AMR genes onto the two-dimensional space characterized by PC1 and PC2.


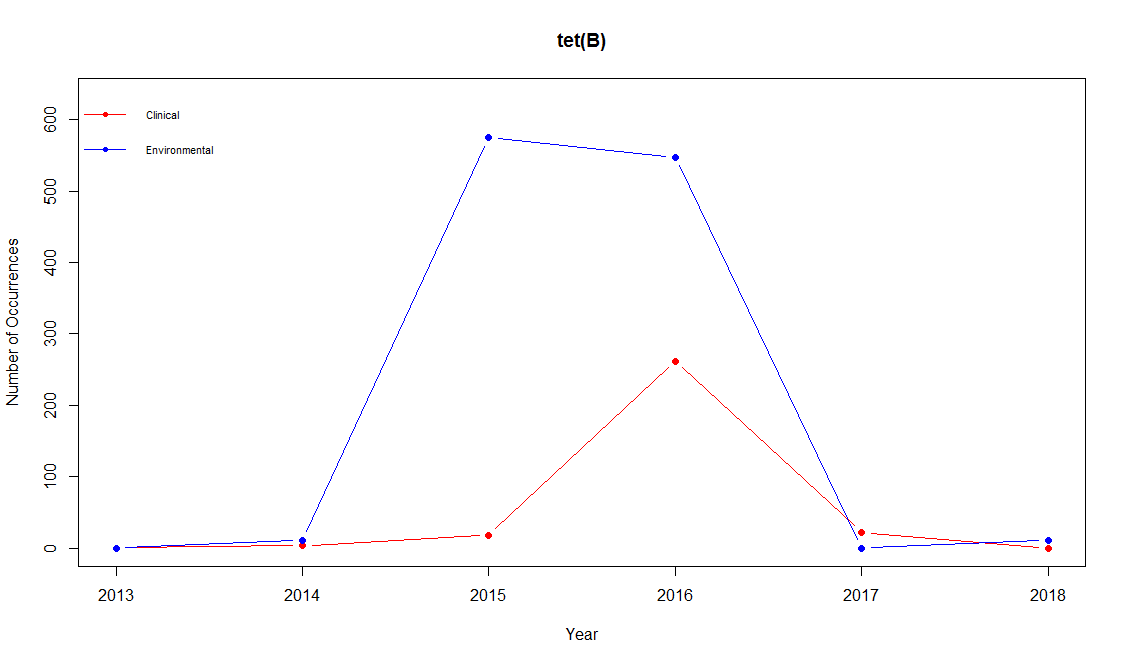

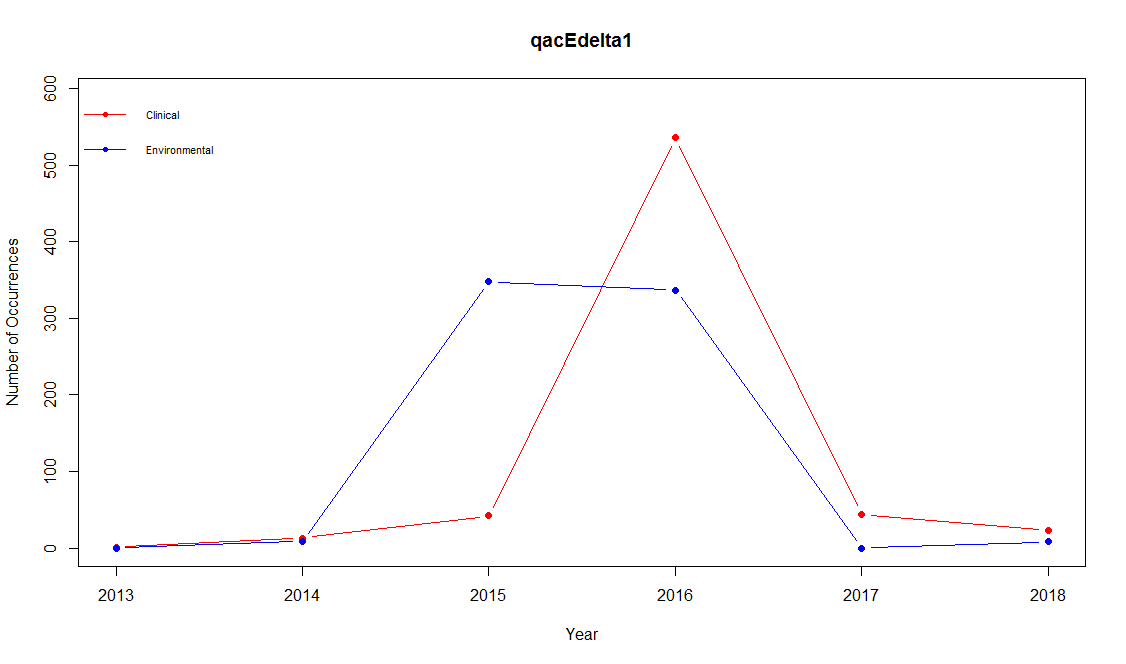


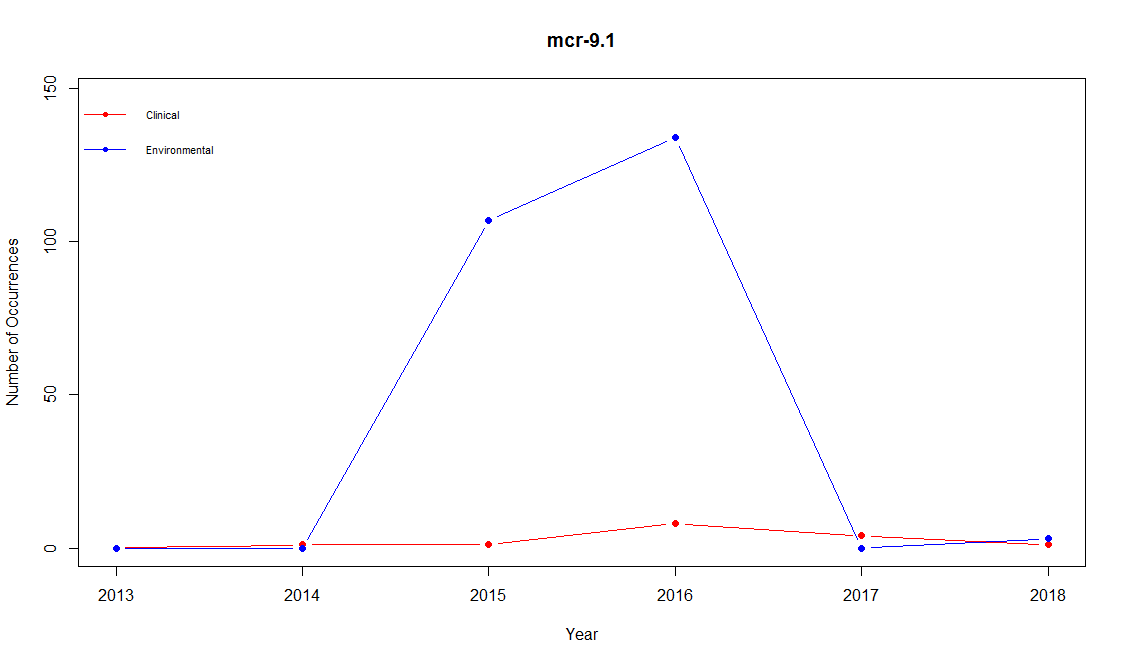

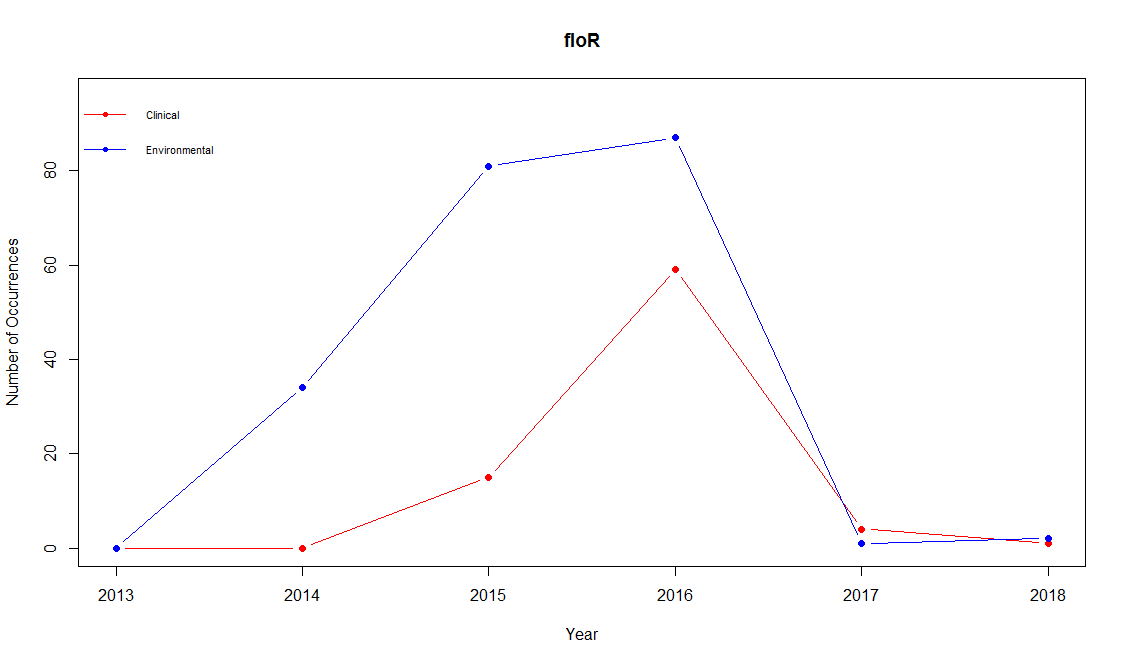


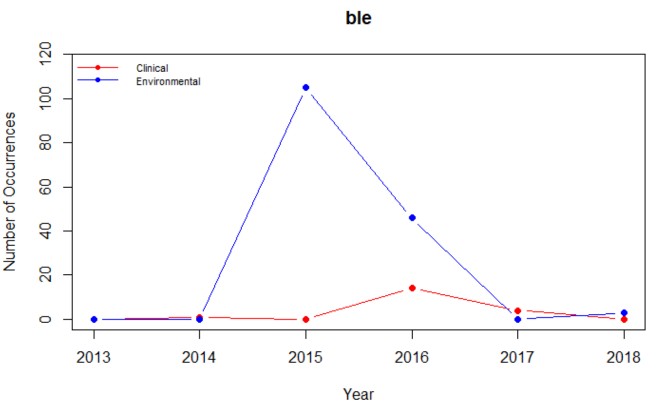

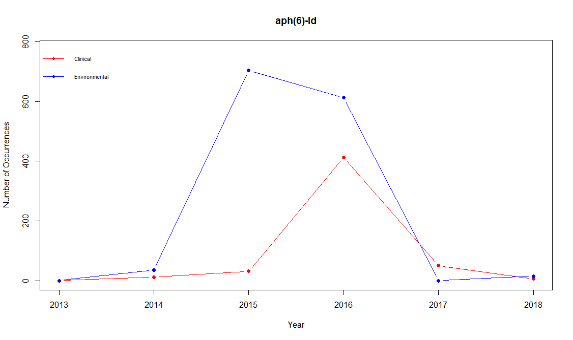

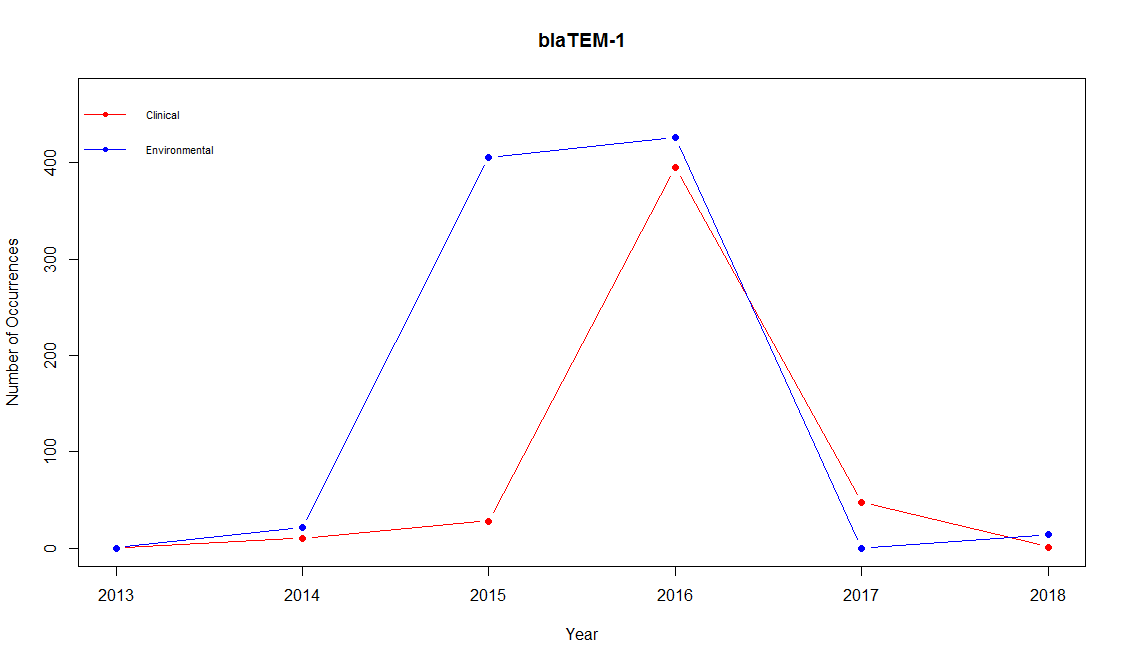

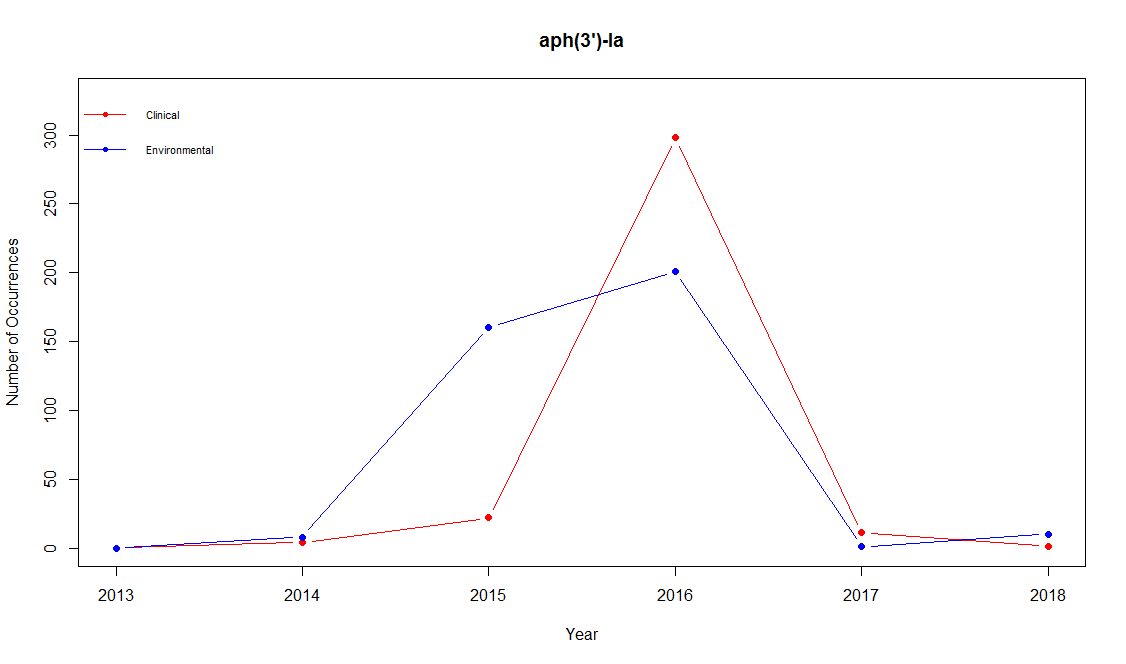


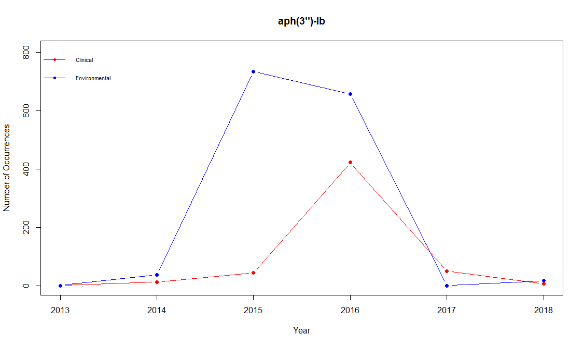

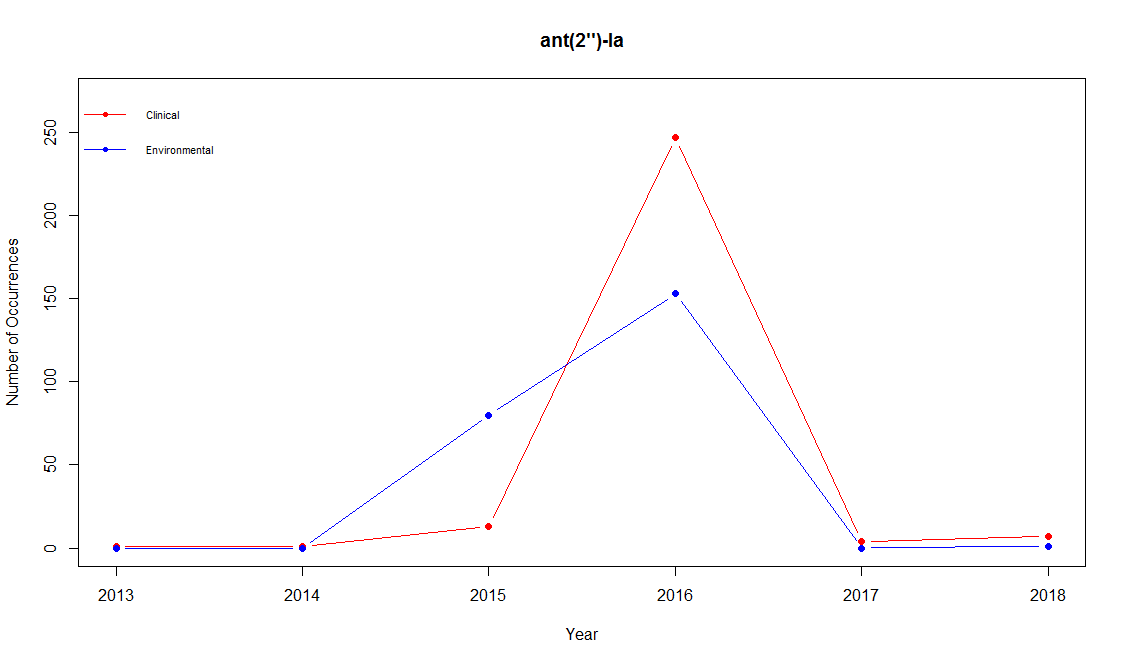


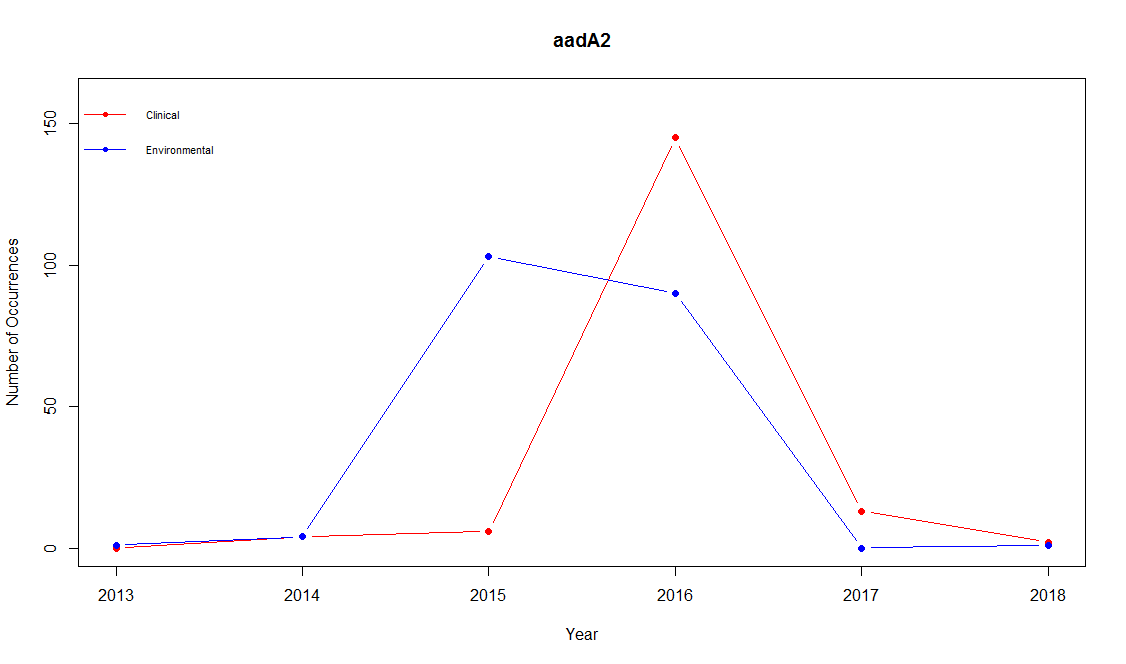

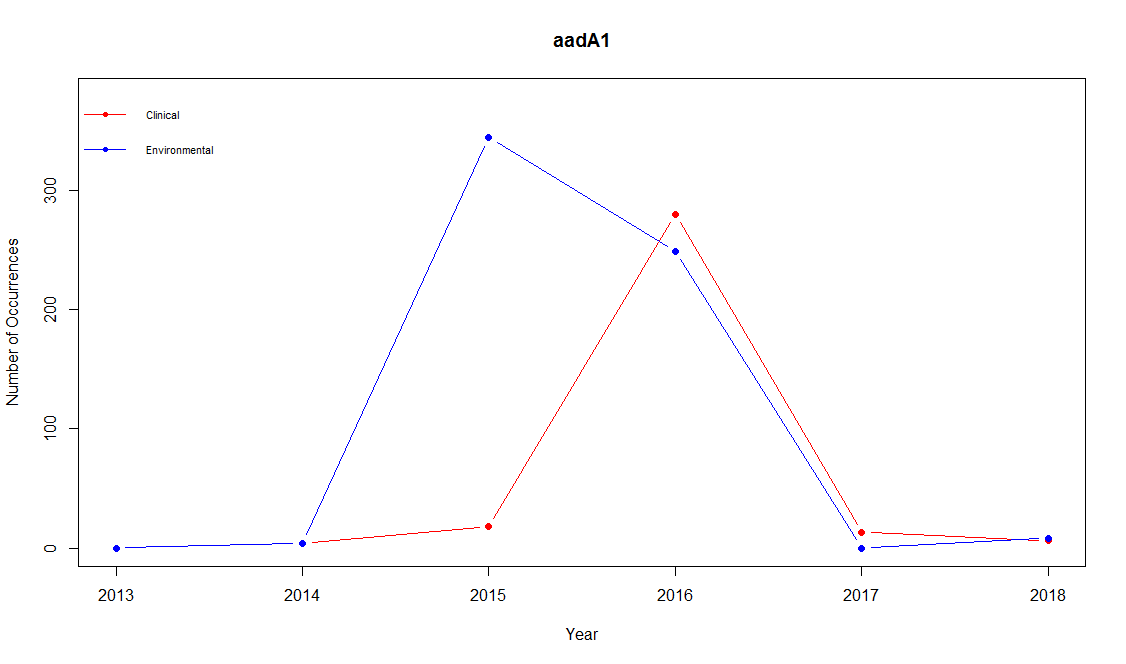


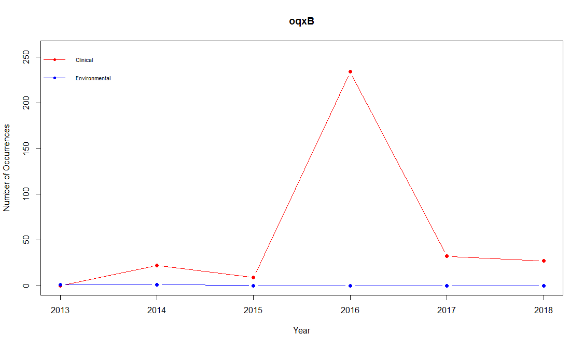

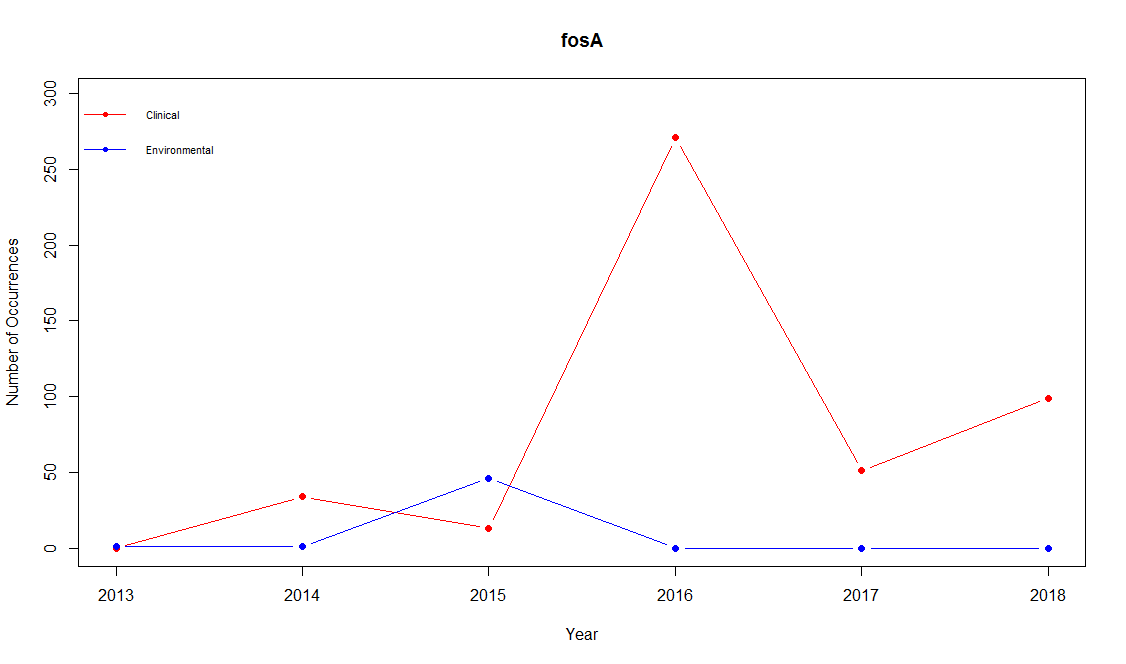

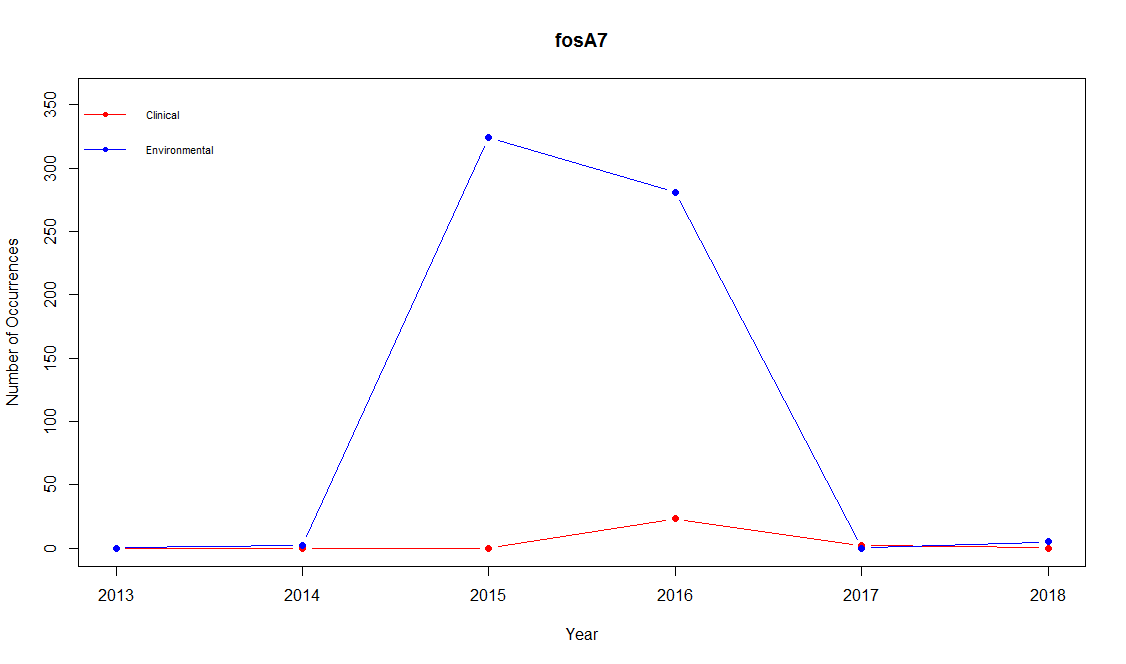


Supplementary Figure 3: the historical occurrence profiles of the other AMR genes (excluding the genes shown in Figure 3).


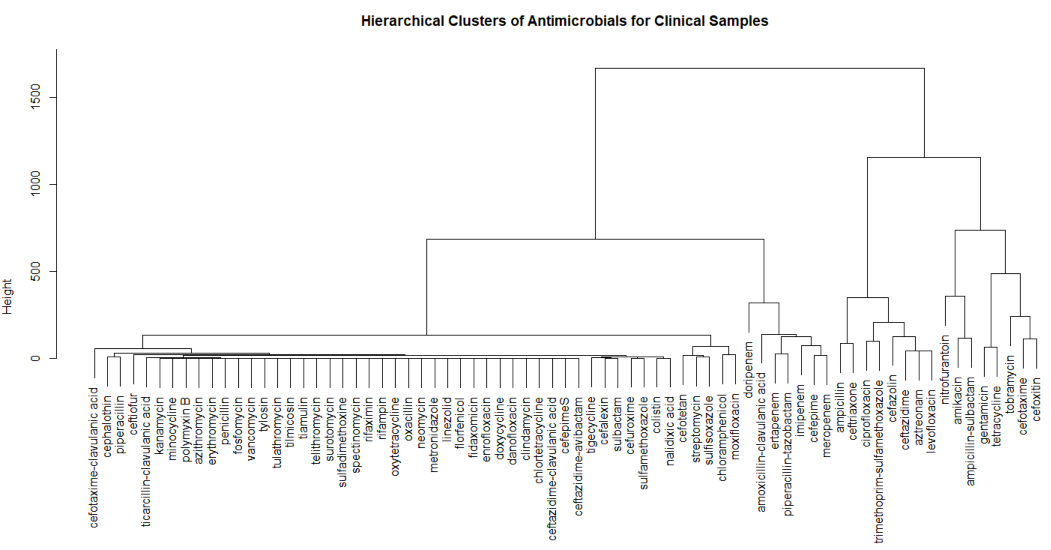


(A)


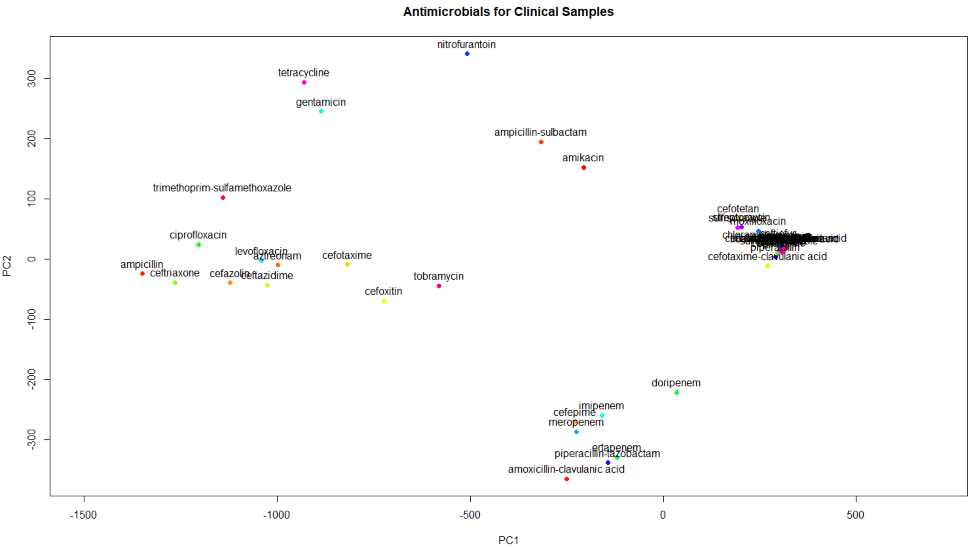


(B)


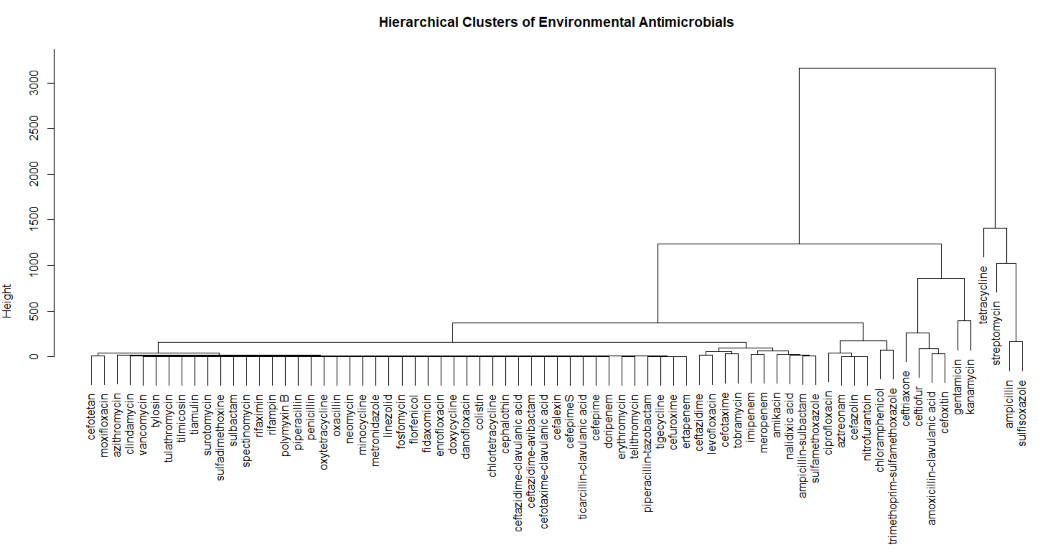


(C)


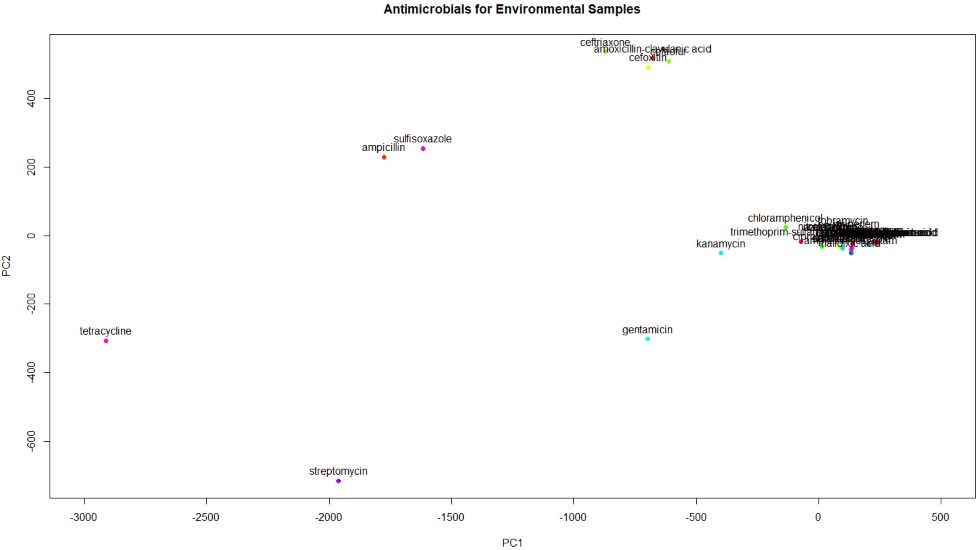


(D)

Supplementary Figure 4: (A) the clustering of antimicrobials resisted by clinical isolates; (B) the projection of clinical antimicrobials onto the two-dimensional space characterized by PC1 and PC2; (C) the clustering of antimicrobials resisted by environmental isolates; (D) the projection of environmental antimicrobials onto the two-dimensional space characterized by PC1 and PC2.


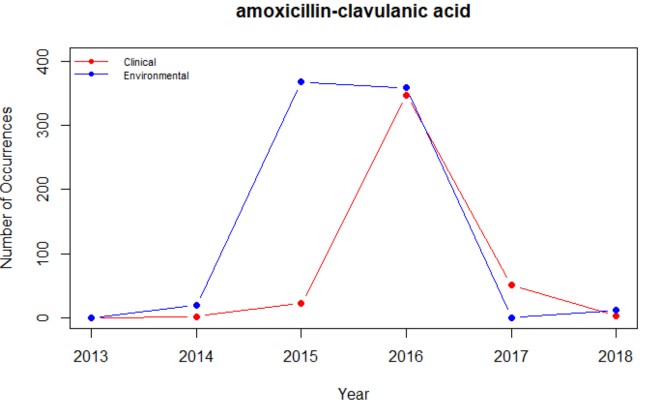

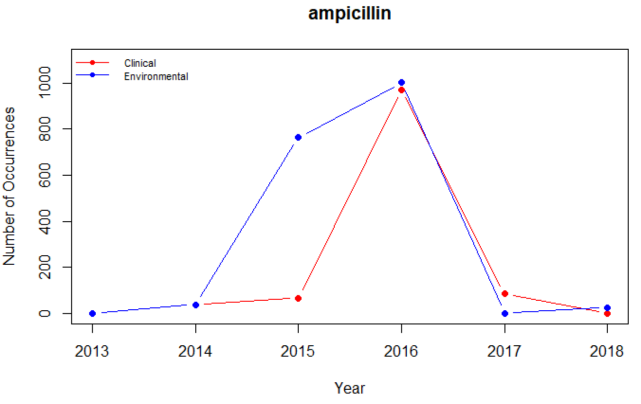


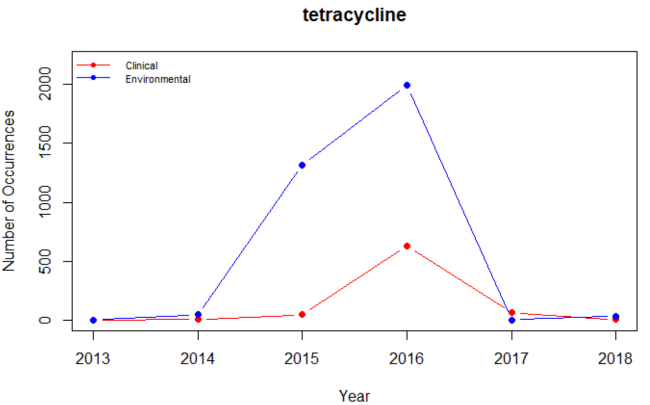

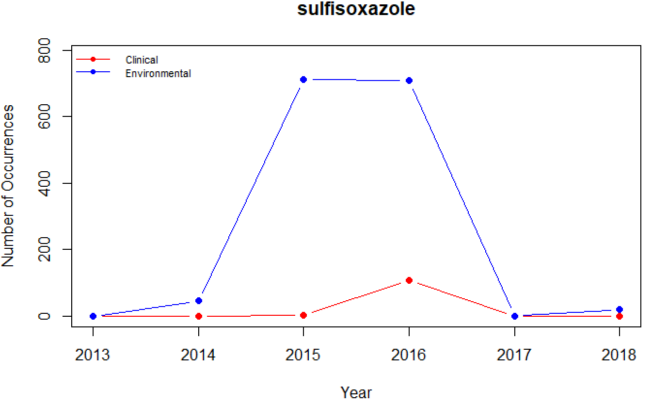


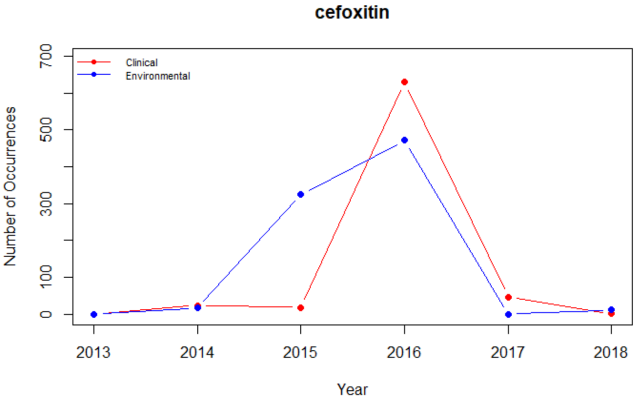

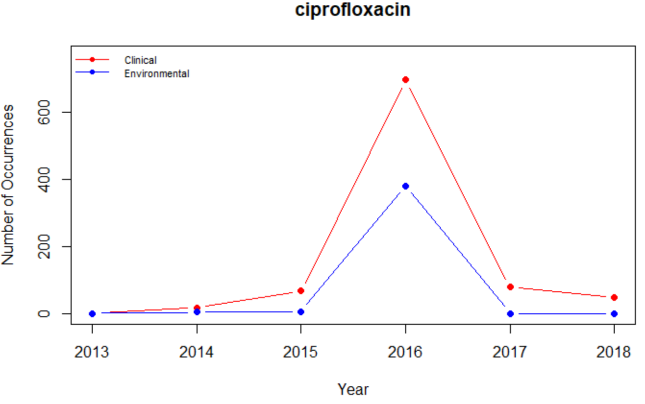


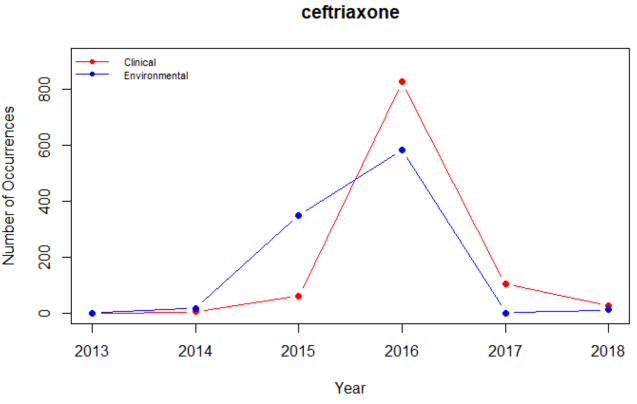

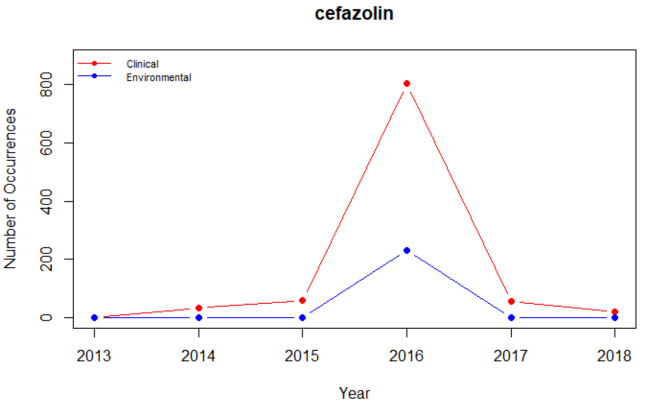


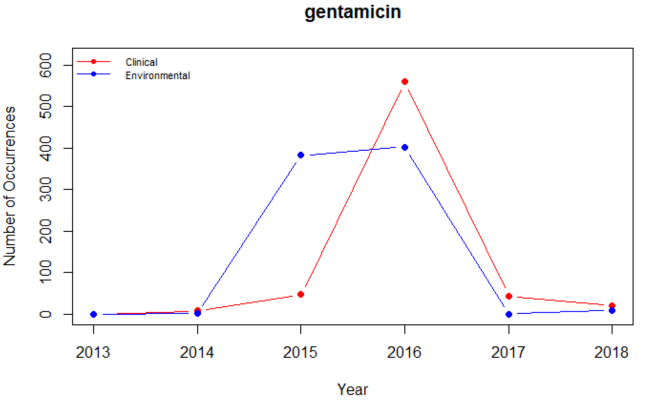

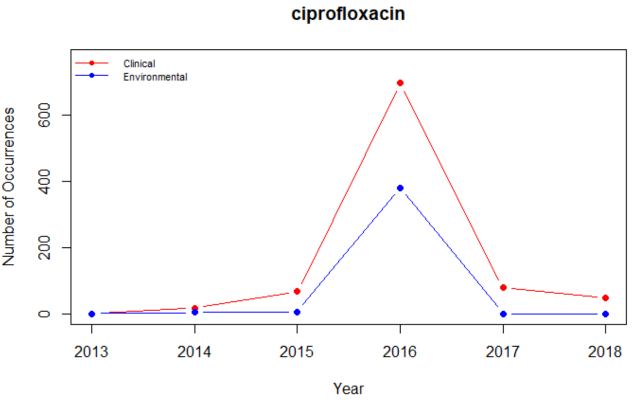


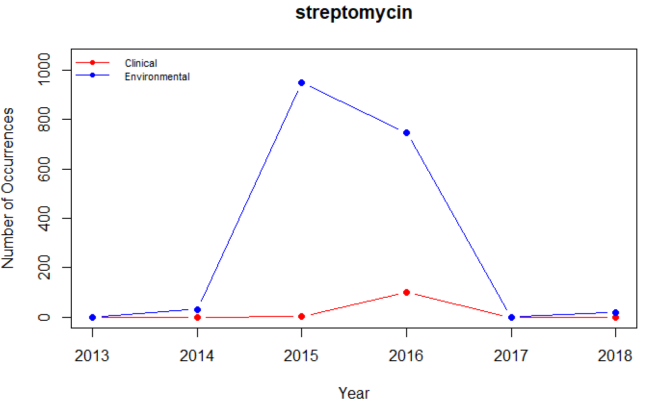


Supplementary Figure 5: the historical occurrence profiles of antimicrobials resisted by clinical and environmental isolates.
